# The Influence of Nucleoside Reverse Transcriptase Inhibitors on Mitochondrial Activity, Lipid Content, and Fatty-Acid-Binding Protein Levels in Microglial HMC3 Cells

**DOI:** 10.3390/ph16121661

**Published:** 2023-11-29

**Authors:** Katarzyna Lipke, Adriana Kubis-Kubiak, Agnieszka Piwowar

**Affiliations:** Department of Toxicology, Faculty of Pharmacy, Wroclaw Medical University, 50-556 Wrocław, Poland

**Keywords:** HIV, NRTIs, fatty acids, cell culture

## Abstract

Despite the availability of a wide range of preventive measures and comprehensive treatment options following infection, the development of acquired immunodeficiency syndrome (AIDS) remains a persistent challenge. Nucleoside reverse transcriptase inhibitors (NRTIs) represent the most commonly utilized therapeutic approach, despite being on the pharmaceutical market for nearly four decades. During this time, a spectrum of side effects ranging from mild discomfort and hypersensitivity reactions to the more prevalent nephrotoxicity and hepatotoxicity has been documented. In light of these considerations, our study aimed to investigate the impacts of two NRTIs, lamivudine and zidovudine, on lipid metabolism in HMC3 microglial cells. Our findings revealed statistically significant reductions in the ATP levels (nearly 8%) and increased mitochondrial superoxide levels (around 10%) after 24 h of treatment with the maximum therapeutic concentration of zidovudine compared to the untreated microglial cells. Furthermore, the concentrations of fatty-acid-binding proteins 4 and 5 were significantly lower (approximately 40%) in the microglial cells that were exposed to NRTIs than in the untreated cells. Notably, the total lipid concentration within the microglial cells markedly increased following NRTI administration with a 13% rise after treatment with 10 µM lamivudine and a remarkable 70% surge following the administration of 6 µM zidovudine. These results suggest that the prolonged administration of NRTIs may potentially lead to lipid accumulation, posing a significant risk to the delicate homeostasis of the neuronal system and potentially triggering a pro-inflammatory response in microglial cells.

## 1. Introduction

Human immunodeficiency virus (HIV) infection poses a pervasive global health challenge, impacting millions of individuals worldwide (UNAIDS, 2021; World Health Organization, 2021). In the several decades since its inception, numerous therapeutic strategies have been developed to mitigate the effects of HIV infection. The primary goal of these therapeutic agents is to curtail the viral load, bolster immune functionality, and elevate the CD4+ cell counts, thereby diminishing the incidence of HIV-related ailments and lowering the risk of viral transmission [1].

Antiretroviral drugs, classified into six groups with distinct mechanisms of action, intervene at various stages of the virus’s replication cycle, thereby hindering its proliferation within the host’s organism. Within the phase of viral replication, reverse transcriptase inhibitors act, and they are further divided into two subgroups. The first subgroup comprises nucleoside reverse transcriptase inhibitors (NRTIs), which competitively inhibit the enzyme by integrating themselves into the DNA chain instead of the substrate for synthesis, leading to premature termination. Notable agents in this group include abacavir, lamivudine (3TC), zidovudine (AZT), and tenofovir. Presently, the conventional therapeutic approach is to amalgamate 2–3 pharmaceuticals of diverse classes, creating highly active antiretroviral therapy (HAART), also known as combined antiretroviral therapy (CART). This regimen has proven to be more efficacious in impeding disease progression compared to monotherapy, extending patient survival by up to 7–10 years [2,3]. This therapeutic approach is suggested to be instituted as early as possible in all HIV-infected individuals, regardless of their CD4+ lymphocyte count. It is imperative to underscore that current antiretroviral therapy does not achieve the complete eradication of the virus from the host’s body. Rather, it plays a pivotal role in retarding the progression of the disease. This is due to the existence of reservoirs housing latent viral forms, sustained viral replication notwithstanding therapeutic interventions, and the virus’s localization within anatomical structures that are relatively inaccessible to pharmaceuticals, including the central nervous system (CNS) [4,5]. NRTIs also exhibit a spectrum of toxic effects, precipitating a multitude of side effects that constitute a significant clinical challenge for patients [3]. These side effects, stemming from nucleoside analogs, can be categorized into those arising from short-term toxicity and long-term toxicity. In the short term, the use of AZT may trigger symptoms such as nausea, vomiting, weakness, or headaches [6,7]. The concomitant administration of 3TC and AZT may induce hypersensitivity reactions (HSR), typified by symptoms including fever, rash, muscle pain, abdominal discomfort, drowsiness, and respiratory distress. Notably, repeated exposure to the drug following HSR may precipitate more severe anaphylactic reactions [8]. Anemia, a common side effect of AZT, results from impaired erythrocyte precursor synthesis in the bone marrow, giving rise to macrocytic anemia. [9]. This circulatory abnormality is primarily associated with thymidine analog drugs, particularly AZT, where macrocytosis is almost universally observed. An elevated mean erythrocyte volume in patients receiving AZT has emerged as an effective marker of treatment efficacy [10]. In the long term, NRTIs induce mitochondrial toxicity primarily via the inhibition of γ polymerase, which is responsible for mitochondrial DNA synthesis. Furthermore, NRTIs inflict oxidative damage, inhibit other mitochondrial enzymes, disrupt ATP synthesis, and trigger cell apoptosis, collectively culminating in the impairment of various systems and organs. The liver is particularly susceptible to mitochondrial toxicity, leading to the inhibition of fatty acid oxidation, ensuing lipid accumulation in the vesicles and transformation into triglycerides. This impedes gluconeogenesis, preventing the conversion of lactic acid into pyruvate, culminating in heightened blood lactate levels. The repercussions encompass symptoms such as malaise, weight loss, rapid breathing, nausea, and vomiting [3]. Patients frequently develop hepatic steatosis and hepatomegaly owing to triglyceride buildup. Severe cases, although relatively rare, may result in liver failure, cardiac arrhythmias, and elevated mortality rates (30–60%) [11]. Notably, among HIV-infected patients, a subtle decline in the bone mineral density is frequently observed, a phenomenon that is likely attributed to the impact of AIDS itself. However, the long-term toxicity of HAART, including NRTIs, significantly exacerbates this condition, ultimately resulting in osteopenia and osteoporosis [4]. AZT administration also contributes to the development of myopathy [12]. The use of NRTIs, coupled with resultant mitochondrial toxicity, induces various metabolic disorders, including disruptions in the lipid metabolism, ultimately giving rise to lipodystrophy—an accumulative fat tissue manifestation, predominantly in the abdominal region—along with, albeit less frequently, lipoatrophy, entailing the irreversible loss of subcutaneous fat tissue. These effects also extend to glucose metabolism due to the induction of insulin resistance [13]. This pertains to the excessive generation of reactive oxygen species, suppressing the expression of genes and proteins involved in pro-adipogenic transcription factors. This results in reduced adipocyte differentiation, accompanied by the impaired metabolism of both fatty acids and glucose [14].

Within the CNS, HIV infection can initiate neuroinflammation and cognitive impairment, collectively referred to as HIV-associated neurocognitive disorders (HANDs) [15,16]. During HIV infection, microglial cells become activated, subsequently releasing inflammatory mediators that contribute to neurotoxicity [17,18]. Notably, HIV proteins such as Tat and the viral envelope protein gp120 have been observed to activate microglial cells, eliciting the release of cytokines, chemokines, and reactive oxygen species (ROS) [19]. The activation of microglial cells during HIV infection has also been associated with the stimulation of the NLRP3 (Nod-like receptor protein family, pyrin domain-containing 3) inflammasome, a multi-protein complex that activates caspase-1 and facilitates the secretion of pro-inflammatory cytokines, including IL-1β and IL-18 [20]. Metabolic perturbations, such as hyperglycemia and dyslipidemia, are commonplace in individuals living with HIV, contributing to the activation of microglial cells [21,22]. Additionally, palmitic acid, a saturated fatty acid, plays a role in promoting inflammation and oxidative stress across various cell types, including microglial cells [23]. The NRTI therapy efficacy in the CNS is constrained by the limited penetration of the blood–brain barrier (BBB) [24]. Furthermore, antiretroviral therapy has varying effects on microglial cells. 3TC mitigates microglial activation and reduces the production of pro-inflammatory cytokines, while AZT does not influence microglial activation but can reduce ROS production.

The research gap in this context lies in the critical need to comprehensively understand the molecular mechanisms underlying microglial activation and the various factors that can induce microglial dysfunction. While the activation of microglial cells during HIV infection is well established, there is a lack of clarity regarding the intricate processes and factors contributing to this activation. This gap in knowledge includes aspects such as the impacts of HIV proteins, metabolic disturbances, and antiretroviral therapy on microglial behavior. To bridge this gap and inform potential therapeutic strategies, our study was designed to investigate the effects of two nucleoside reverse transcriptase inhibitors (NRTIs), 3TC and AZT, on HMC3 microglial cells’ homeostasis. By examining key intracellular parameters, including the ATP levels, ROS production, lipid content, and the expression of fatty-acid-binding proteins (FABP4 and FABP5), our research aims to provide fresh insights into how these molecules may function in the context of HIV infection within the CNS. This research is essential for the development of more effective treatment approaches for HIV and the prevention of HANDs. Furthermore, our findings have the potential to offer fresh insights into the mechanisms of action of these molecules in the treatment of HIV infection within the CNS.

## 2. Results

The HMC3 cell line, derived from transformed human microglial cells, faithfully maintains the essential characteristics of primary microglial cells. These cells form a homogeneous and nearly immortal population that is suitable for in-depth biochemical investigations into the functions of microglial cells. In addition to this, HMC3 cells demonstrate an amenability to transfection, providing a valuable platform for the study of gene regulation within microglial cells. Originating from primary human fetal brain-derived microglia, HMC3 cells display a resting state phenotype that includes a strong expression of the microglia/macrophage marker IBA1 and the endotoxin receptor CD14. Notably, they do not express the astrocyte marker GFAP. The transition to an activated microglial state, indicated by the upregulation of markers such as MHCII, CD68, and CD11b, can be achieved by exposure to IFN-gamma, highlighting their suitability for modelling activated microglia in studies. HMC3 cells are a valuable and adaptable resource for researchers investigating the intricacies of microglial cell function, particularly in the context of neuroinflammation and neurological disorders.

### 2.1. Cell Cytotoxicity Assay: XTT

**Figure 1:**
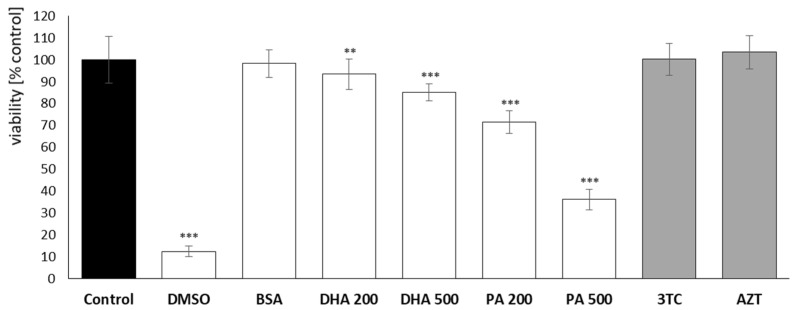
Cytotoxic effect of 24 h incubation with DHA, PA, 3TC, or AZT on microglial cells. Control—untreated HMC3 cells; DMSO—positive control, cells treated with 10% DMSO; BSA—incubation with 10% fatty acid-free BSA; DHA200 and DHA 500—incubation with 200 or 500 µM DHA; PA200 and PA 500—incubation with 200 or 500 µM PA; 3TC—incubation with 10 µM lamivudine; AZT—incubation with 6 µM zidovudine. Statistically significant differences compared to the control. ** *p* < 0.01; *** *p* < 0.001.

The impact of the investigated NRTIs on microglial cell viability was evaluated using the XTT assay. The findings following a 24 h incubation period with DHA, PA, and NRTIs are depicted in Figure 1 and are presented as a percentage of viability relative to the control group (unexposed cells). As a positive control for measuring cytotoxic effects, incubation with 10% DMSO was employed.

As anticipated, the most pronounced cytotoxicity was evident in the microglial cells following the introduction of 200 µM and 500 µM PA, resulting in cell mortalities of approximately 28% and 64%, respectively. An evaluation of the impact of docosahexaenoic acid (DHA), representing polyunsaturated omega-3 fatty acids, did not demonstrate a substantial adverse effect on the HMC3 cell viability, even at lipotoxic concentrations, with a mortality rate of approximately 15%. Notably, no indications of cytotoxicity were observed following a 24 h incubation with NRTIs.

### 2.2. ATP Measurement Assay, Bioluminescence Assay Kit HS II

**Figure 2:**
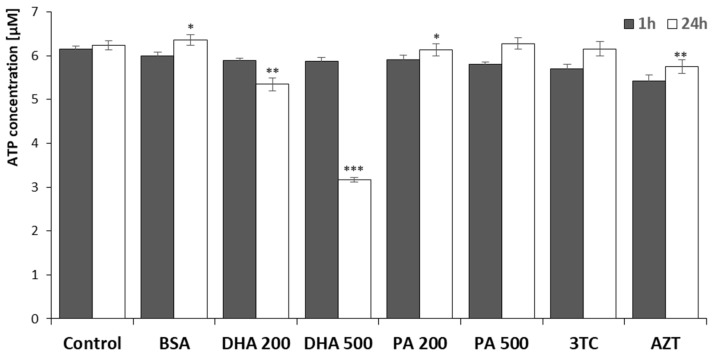
Mitochondrial ATP levels after 1 h and 24 h incubation with DHA, PA, 3TC, or AZT. Control—untreated HMC3 cells; BSA—incubation with 10% fatty acid-free BSA; DHA200 and DHA 500—incubation with 200 or 500 µM DHA; PA200 and PA500—incubation with 200 or 500 µM PA; 3TC—incubation with 10 µM lamivudine; AZT—incubation with 6 µM zidovudine. Statistically significant differences compared to the untreated HMC3 cells: * *p* < 0.05; ** *p* < 0.01; *** *p* < 0.001.

The quantification of ATP levels following 1 h and 24 h of incubation with DHA, PA, or the evaluated NRTIs is depicted in Figure 2.

The initial analysis, involving a 1 h incubation period and focusing on the impact of NRTIs on the ATP levels, did not reveal significant differences. However, following a 24 h incubation, certain compounds exhibited a notable effect on ATP activation, resulting in statistically significant reductions in the ATP concentrations in specific instances. The most substantial influence was observed after incubation with DHA at concentrations of 200 µM and 500 µM, leading to decreases of 15% and 50% (*p* < 0.01 and *p* < 0.001, respectively). Additionally, a nearly 8% decrease was recorded after the administration of 6 µM of zidovudine. Furthermore, a statistically significant reduction in the ATP levels (in comparison to the untreated cells) was also observed after a 24 h incubation with 200 µM of PA.

### 2.3. Mitochondrial Superoxide Levels, MitoSOX^TM^ Red Assay

**Figure 3:**
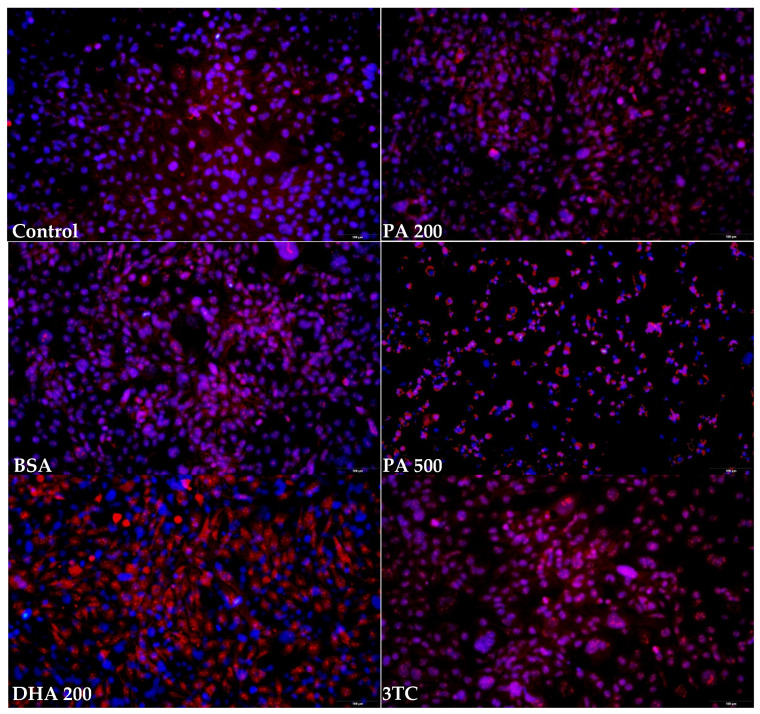

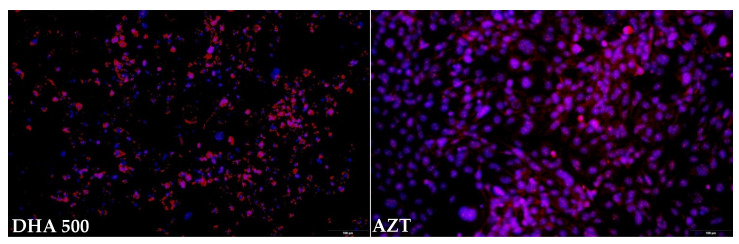
Examples of fluorescence microphotographs of fixed HMC3 cells stained with MitoSOX (red) and counterstained with DAPI (blue). Magnification: 20×. Control—untreated HMC3 cells; BSA—incubation with 10% fatty acid-free BSA; DHA200 and DHA500—incubation with 200 or 500 µM DHA; PA200 and PA500—incubation with 200 or 500 µM PA; 3TC—incubation with 10 µM lamivudine; AZT—incubation with 6 µM zidovudine.

Mitochondrial superoxide production can be visualized through fluorescence microscopy by employing the MitoSOX superoxide indicator. MitoSOX is particularly susceptible to rapid oxidation via superoxide, distinguishing it from other reactive oxygen species and reactive nitrogen species. The results of the fluorescence analysis are illustrated in Figure 3. 

An examination of the fluorescence micrographs revealed that the treatment with 500 µM DHA and 500 µM PA resulted in a distinct punctate fluorescence pattern, distinguishing it from the outcomes observed in the other experimental conditions. Conversely, the staining appeared more diffused and blurred in the case of 3TC and AZT when compared to the control cells. To quantitatively assess the differences between the various treatments, the MitoSOX fluorescence was spectrophotometrically measured at 580 nm.

**Figure 4:**
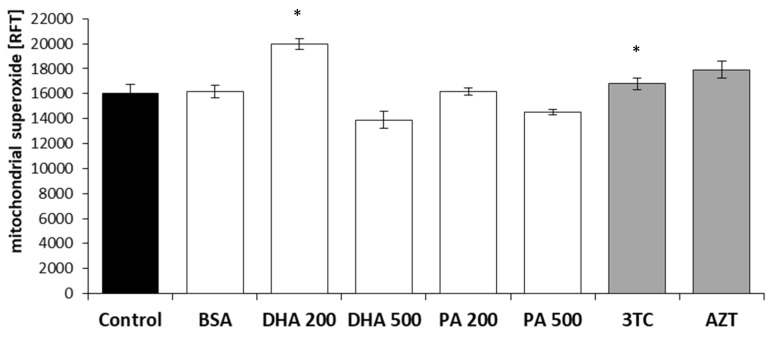
Mitochondrial superoxide levels after 24 h incubation with DHA, PA, 3TC, or AZT. Control—untreated HMC3 cells; BSA—incubation with 10% fatty acid-free BSA; DHA200 and DHA500—incubation with 200 or 500 µM DHA; PA200 and PA500—incubation with 200 or 500 µM PA; 3TC—incubation with 10 µM lamivudine; AZT—incubation with 6 µM zidovudine. Statistically significant differences compared to the untreated HMC3 cells: * *p* < 0.05.

The quantification of the mitochondrial superoxide levels was conducted in methanol-fixed HMC3 cells following a 24 h incubation with DHA, PA, or the tested NRTIs, as demonstrated in Figure 4.

The addition of 200 µM of DHA exhibited a statistically significant increase of approximately 25% in the detectable MitoSOX fluorescence. In the case of NRTI administration, we anticipated an elevation in the mitochondrial superoxide of around 10%, with statistical significance being achieved in the case of 3TC. Following incubation with the lipotoxic concentrations of DHA and PA, a reduction in the MitoSOX signal of nearly 10% and 13%, respectively, was observed.

### 2.4. The FABP 4 and 5 Concentrations, Human ELISA

**Figure 5:**
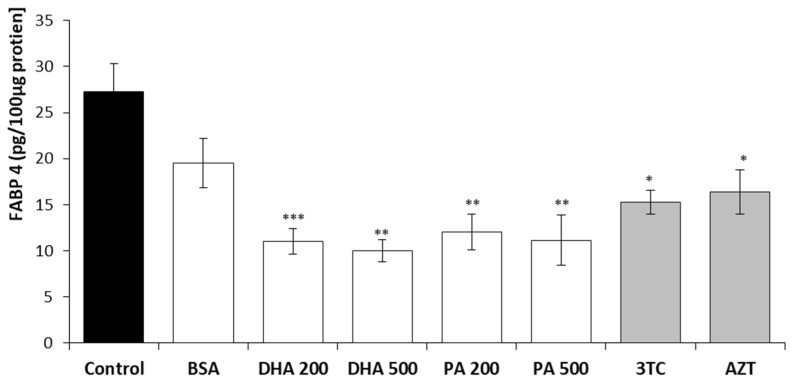
Intracellular FABP4 levels after 24 h incubation with DHA, PA, 3TC, or AZT. Control—untreated HMC3 cells; BSA—incubation with 10% fatty acid-free BSA; DHA200 and DHA 500—incubation with 200 or 500 µM DHA; PA200 and PA 500—incubation with 200 or 500 µM PA; 3TC10—incubation with 10 µM lamivudine; AZT6—incubation with 6 µM zidovudine. Statistically significant differences compared to the untreated HMC3 cells: * *p* < 0.05, ** *p* < 0.01, *** *p* < 0.001.

**Figure 6:**
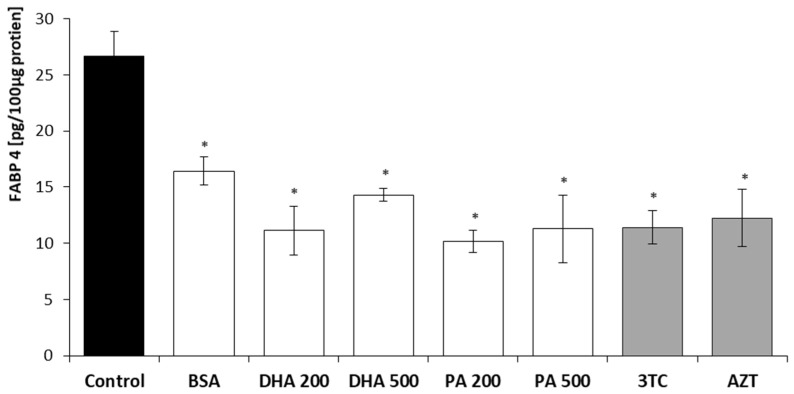
Intracellular FABP5 levels after 24 h incubation with DHA, PA, 3TC, or AZT. Control—untreated HMC3 cells; BSA—incubation with 10% fatty acid-free BSA; DHA200 and DHA 500—incubation with 200 or 500 µM DHA; PA200 and PA 500—incubation with 200 or 500 µM PA; 3TC 10—incubation with 10 µM lamivudine; AZT 6—incubation with 6 µM zidovudine. Statistically significant differences compared to the untreated HMC3 cells: * *p* < 0.05.

The levels of FABP4 and FABP5 in HMC3 cell lysates following a 24 h incubation with DHA, PA, or the tested NRTIs are depicted in Figure 5 and Figure 6. The results were normalized to a total protein concentration of 100 µg in the cell lysates, as determined using the BCA protein assay.

The intracellular levels of the FABP4 transporter exhibited a reduction in all experimental conditions, with the lowest values observed for the lipotoxic concentrations of 500 µM DHA and 500 µM PA, corresponding to 36% and 40% of the control, respectively. The physiological concentrations of 200 µM DHA or 200 µM PA showed slightly higher intracellular FABP4 levels. Incubation with 10 µM lamivudine or 6 µM zidovudine also led to a decrease of approximately 40%. Additionally, the results obtained after the administration of the carrier for polyunsaturated and unsaturated fatty acids (BSA) exhibited a decrease of 28% compared to the untreated cells, although this decrease was not statistically significant.

Similar to the observations with FABP4, the intracellular levels of the FABP5 transporter were reduced in all of the experimental conditions. The lowest levels of FABP5 were measured after the treatment with 200 µM DHA and 200 µM PA, resulting in a decrease of approximately 60%, while the lipotoxic concentrations of 500 µM DHA and 500 µM PA caused higher levels of FABP5, with concentration levels of 53% and 42% compared to the untreated cells. Incubation for 24 h with 10 µM of lamivudine or 6 µM of zidovudine led to similar results as those obtained for 500 µM of PA.

### 2.5. MAPK Family Activation Measurement, InstantOne ELISA™

**Figure 7:**
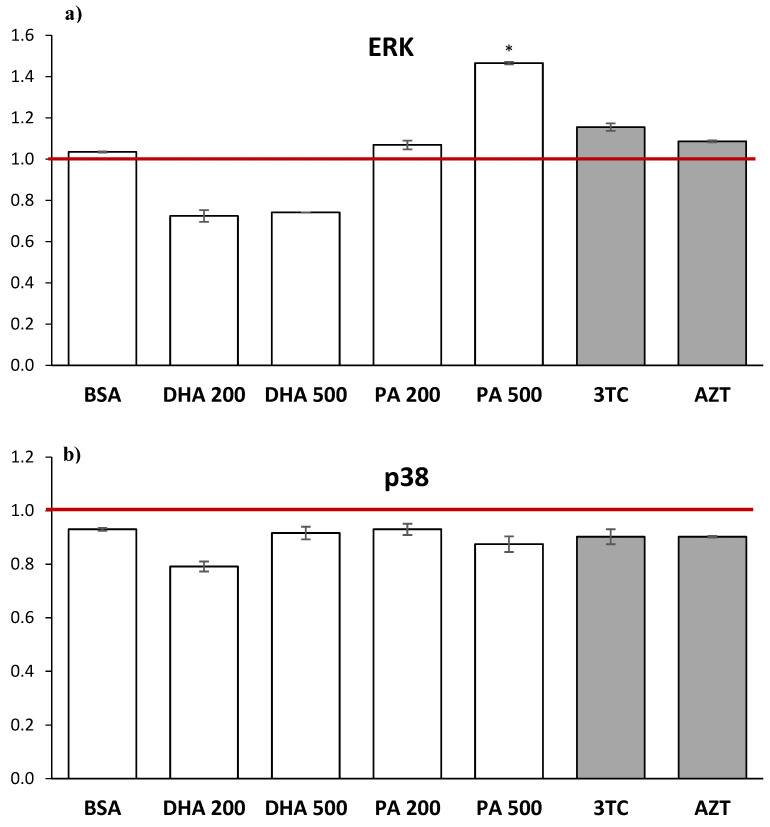

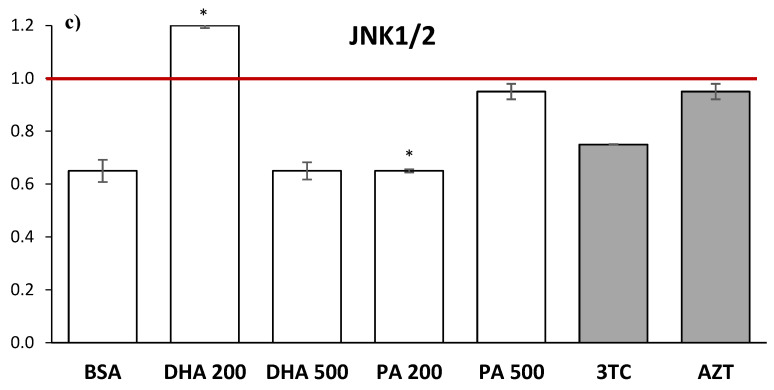
Intracellular ERK, p38, and JNK1/2 phosphorylation levels after 24 h incubation with DHA, PA, 3TC, or AZT. The outcomes are displayed as multiples of the data acquired from the control, where the untreated HMC3 cells are established as having a value of 1 (red line). Control—untreated HMC3 cells; BSA—incubation with 10% fatty acid-free BSA; DHA200 and DHA 500—incubation with 200 or 500 µM DHA; PA200 and PA 500—incubation with 200 or 500 µM PA; 3TC—incubation with 10 µM lamivudine; AZT—incubation with 6 µM zidovudine. Statistically significant differences compared to the untreated HMC3 cells: * *p* < 0.05,.

The phosphorylation levels of the ERK, p38, and JNK1/2 proteins were analyzed in the HMC3 cell lysates following a 24 h incubation with DHA, PA, or the tested NRTIs. The results in Figure 7 are presented as multiples of the data obtained for the control, with the value for the untreated HMC3 cells (control) set as 1.

As depicted in Figure 7a, it appears that only high levels of PA (500 µM) cause a statistically significant activation of intracellular ERK phosphorylation, with an approximately 1.5-fold increase compared to the control. Additionally, a 24 h treatment with 10 µM of lamivudine resulted in the activation of intracellular ERK phosphorylation (approximately 1.15-fold compared to the control), although statistical significance was not achieved. When assessing the intracellular p38 protein phosphorylation levels, the administration of all substances led to a reduction in this process. The addition of NRTIs to HMC3 cells for 24 h did not significantly affect this process. In contrast, statistically significant increases in the intracellular JNK1/2 phosphorylation levels, compared to the untreated cells, were observed after the treatment with physiological levels of DHA (approximately 1.2-fold), while the same concentrations of PA led to the inhibition of this process (approximately 0.65-fold). By analyzing the effect of NRTIs on intracellular JNK1/2 activation, it appears that 10 µM of lamivudine inhibits this process (approximately 0.75-fold), although statistical significance was not achieved.

### 2.6. Lipid Concentration Analysis, Oil Red O Staining 

**Figure 8:**
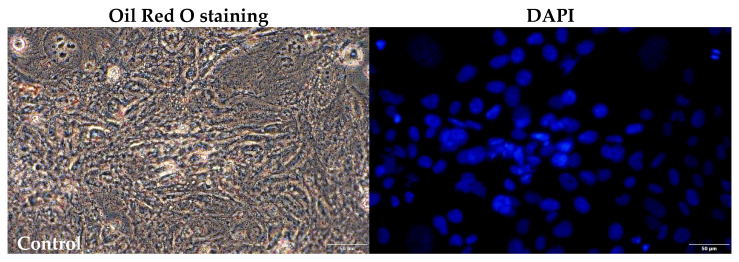

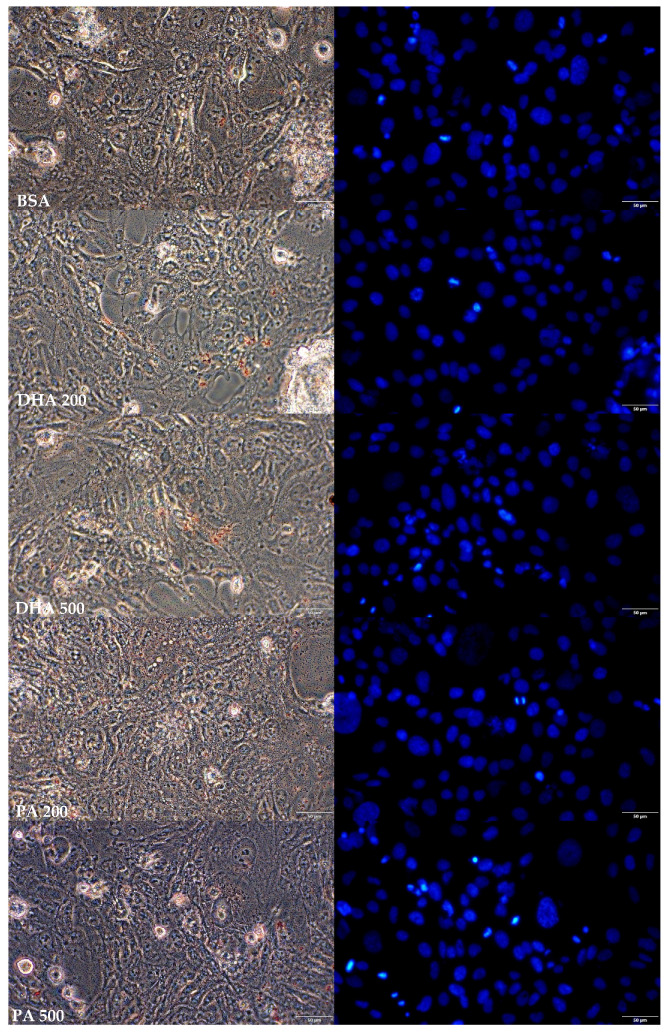

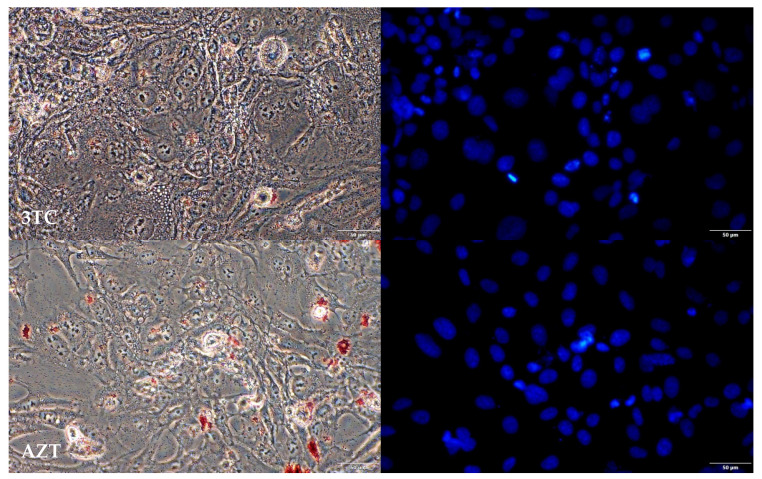
Examples of fluorescence microphotographs of fixed HMC3 cells stained with Oil Red O (red) and DAPI (blue). Magnification: 20×. Control—untreated HMC3 cells; BSA—incubation with 10% fatty acid-free BSA; DHA200 and DHA 500—incubation with 200 or 500 µM DHA; PA200 and PA 500—incubation with 200 or 500 µM PA; 3TC 10—incubation with 10 µM lamivudine; AZT 6—incubation with 6 µM zidovudine.

**Figure 9:**
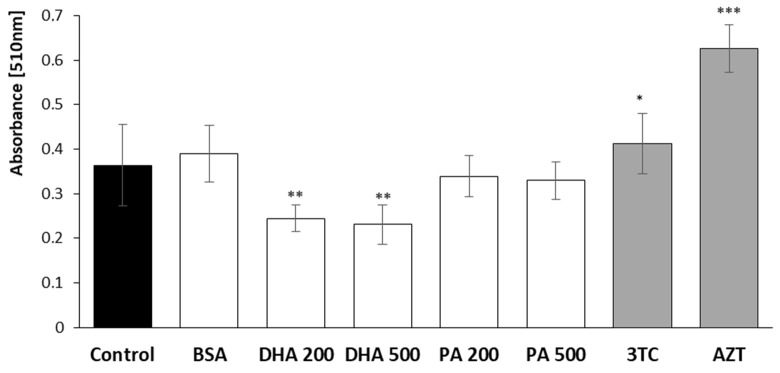
Quantification of the stained lipid performed using the eluted Oil Red O stain via measuring absorbance at 510 nm. Control—untreated HMC3 cells; BSA—incubation with 10% fatty acid-free BSA; DHA200 and DHA 500—incubation with 200 or 500 µM DHA; PA200 and PA 500—incubation with 200 or 500 µM PA; 3TC 10—incubation with 10 µM lamivudine; AZT 6—incubation with 6 µM zidovudine. Statistically significant differences compared to the untreated HMC3 cells: * *p* < 0.05, ** *p* < 0.01, *** *p* < 0.001.

A microscopic evaluation of triglyceride staining and quantification was performed in PFA-fixed HMC3 cells after 24 h of incubation with DHA, PA, or the tested NRTIs. The results are presented in Figure 8 and Figure 9.

The lipid content was determined through Oil Red O staining, and quantification of the stained lipid was achieved by measuring the absorbance at 510 nm. Interestingly, we observed a contrasting effect of the DHA on the lipid content in the HMC3 cells compared to the results obtained after 24 h of incubation with the NRTIs. Both concentrations of DHA led to a decrease in the lipid content, with a reduction of 32% observed at the physiological concentration and a reduction of 36% following the application of lipotoxic levels. In contrast, incubation with NRTIs for 24 h resulted in an increase in the lipid quantity, with a 13% rise after the treatment with 10 µM lamivudine and a remarkable 70% surge following the administration of 6 µM of zidovudine. Notably, there were no observable effects when the physiological nor lipotoxic concentrations of PA were added.

## 3. Discussion

In the context of the brain, lipids play multifaceted roles, including the involvement in brain development, neurogenesis, synaptogenesis, myelin sheath formation, and signaling processes. The dysregulation of lipid homeostasis is associated with brain damage, as well as various metabolic and neurological disorders. The term “lipotoxicity” refers to cellular damage resulting from an excess of free fatty acids (FFAs). Palmitic acid (PA), the most prevalent saturated fatty acid in the bloodstream, has been shown to induce apoptotic cell death in numerous cell types, including neonatal rat myocytes, pancreatic β cells, skeletal muscle cells, liver cells, podocytes, and hypothalamic neurons. Elevated levels of FFAs have been implicated as a potential risk factor for Alzheimer’s disease, particularly in neurons and astrocytes, possibly contributing to cognitive impairment. The specific lipotoxic effects of PA on nerve cells remain an area of limited research. Furthermore, there is a knowledge gap concerning the mechanisms through which docosahexaenoic acid (DHA) safeguards cells against lipotoxicity [25] Many studies investigating the lipotoxic properties of NRTIs primarily focus on visceral tissues. Consequently, their effects on the human brain remain largely unexplored, giving rise to several unanswered questions and hypotheses [25,26,27,28].

Zidovudine, also known as 3-azido-3′-deoxythymidine, was the first NRTI to be approved for antiretroviral therapy. Initially approved for adults in 1987 and later for children in 1990, AZT exhibits activity against HIV-1 and HIV-2, as well as against other retroviruses like Spumavirinae, Lentivirinae, and Oncovirinae. AZT, a thymine analogue, competes with thymidine during DNA strand extension. Following oral administration, it is rapidly absorbed in the gastrointestinal tract, achieving peak serum concentrations within approximately 0.5 to 1.5 h, with an average bioavailability of 64%. The drug is widely distributed throughout the body, owing to its lipophilic nature, enabling excellent penetration into the CNS and genital secretions. Its plasma protein binding typically remains below 38%. Metabolized in the liver, the majority of AZT is excreted in the form of glucuronide in the urine, with only 14–18% of that excreted being unchanged. Lamivudine, often referred to as 2′-deoxy-3′-thiacytidine (3TC), is a hydrophilic analogue of cytidine, serving not only as an agent against HIV but also as a component of therapy for Hepatitis B Virus. Orally administered, 3TC is well absorbed in the gastrointestinal tract, reaching peak serum concentrations in a similar timeframe to AZT. Its bioavailability surpasses that of AZT, reaching 82–88% in adults and 66–68% in children, with a plasma protein binding rate of 38%. While 3TC penetrates the CNS to a lesser extent (about 5–10% of plasma concentration), it accumulates notably in semen and cervicovaginal secretions. Approximately 70% of the drug that is excreted in the urine is unchanged, with around 5% being excreted as a sulfoxide derivative. It is worth noting that 3TC is not recommended for monotherapy use due to the frequent development of drug resistance within the first three months of treatment, leading to reverse transcriptase mutations [6,29,30].

This investigation delved into the impact of the maximum therapeutic concentrations of two NRTIs, namely 3TC and AZT, on microglial cells. Utilizing the XTT assay, we did not find evidence of cytotoxic effects, as the cell viability closely paralleled that of the control sample. This finding aligns with the earlier research by Akay et al. [3], who did not observe a significant decrease in the survival of neuroglial cells that were exposed to AZT for 48 h. Additionally, Hung et al. [16] examined the effects of NRTIs (AZT, didanosine, tenofovir, and emtricitabine) on the survival of neuronal cells in the cerebral cortex. They reported that at the fifth day of evaluation, the cytotoxicity measurements resembled those of the control, while longer incubation periods (10 and 14 days) revealed substantial cytotoxic effects. This underscores the potential impact of the exposure duration of a given compound on its effects.

Mitochondrial toxicity is recognized as a prominent adverse effect of nucleoside analog treatment. To further explore these effects, we evaluated the ATP concentrations and superoxide production in HMC3 cells following the exposure to 3TC and AZT. A brief one-hour incubation did not yield any discernible differences in the ATP levels. However, after a 24 h exposure, we observed statistically significant reductions in the ATP levels following incubation with 6 µM of AZT. Moreover, the 24 h treatment with NRTIs resulted in heightened levels of mitochondrial superoxide compared to the untreated cells. These observations might be linked to the activation of pathological mitochondrial pathways, initiating from mitochondrial DNA inhibition and extending to the production and accumulation of dysfunctional proteins, impaired fatty acid oxidation, and compromised oxidative phosphorylation, and culminating in an increased production of reactive oxygen species due to electron leakage from the electron transport chain. This deficit impacts ATP production and, consequently, leads to inadequate energy levels to maintain cellular homeostasis, potentially resulting in the death of microglial cells. The existing findings underscore that the duration of NRTI use and the use of NRTIs with substantial mitochondrial DNA inhibition constitute significant risk factors for lipoatrophy development [31].

Furthermore, we assessed the impacts of NRTIs on the levels of two fatty-acid-binding proteins, namely FABP4 and FABP5, in HMC3 cells. Following the exposure to both 3TC and AZT, we observed substantially lower concentrations of FABP4 compared to the control cells. FABP4 is known to play a role in inhibiting lipogenesis while promoting lipolysis, thereby influencing the composition of circulating free fatty acids [32]. Additionally, it significantly contributes to the promotion of lipotoxicity, mainly by triggering endoplasmic reticulum stress and oxidative stress through its actions on the mitochondria, leading to reactive oxygen species production and subsequent inflammation. Consequently, an increase in the FABP4 levels may indicate the presence of metabolic disorders [33]. Much of the research conducted thus far has focused on disorders of visceral and subcutaneous adipose tissue in association with NRTI use. For instance, Boothby et al. [6] measured FABP4 expression in patients undergoing various combinations of antiretroviral drugs over a six-month period and reported an approximately 2.5-fold increase in the FABP4 expression in patients receiving a combination of 3TC and AZT with a non-nucleoside reverse transcriptase inhibitor (NNRTI). Similarly, Escoté et al. [34] measured FABP4 in patients undergoing antiretroviral therapy, including prolonged stavudine treatment, and noted a significant correlation between increased FABP4 levels and the occurrence of lipodystrophy. Regarding FABP5, this protein is not exclusively associated with adipocytes and is expressed across various cell types. Researchers have suggested its involvement in the uptake of DHA by endothelial cells in brain blood vessels, playing a crucial role in preserving cognitive functions. Therefore, alterations in FABP5 expression may have an impact on the development of neurocognitive symptoms [35,36,37]. In our study, we observed a significant decrease in the FABP5 concentrations after exposing HMC3 cells to 3TC and AZT. Following a statistical analysis, both sets of results indicated these changes as statistically significant. At present, no studies have investigated the effects of NRTI drugs on FABP5 concentrations, making our findings a valuable reference point for future research. Furthermore, we found that 3TC and AZT had no discernible effects on the phosphorylation pathways of ERK1/2, p38, and JNK1/2, at least within the scope of our experimental settings. It might be worth exploring whether these compounds influence the genes that are responsible for activating the MAPK family. Importantly, we noted a considerable increase in the total lipid concentrations in the HMC3 cells following 3TC or AZT administration, supporting the notion that long-term NRTI use could potentially lead to lipid accumulation. This could pose a significant risk to the delicate homeostasis of the neuronal system and potentially trigger a pro-inflammatory response.

There is a limited body of literature addressing the impact of NRTIs on microglial functions within the brain. In a study conducted by Giunta et al. [38], the effects of various ART, including 3TC and AZT, on the microglial capacity to clear Aβ and potentially exacerbate amyloidosis were investigated. The results indicated a significant hindrance of the microglial phagocytosis of FITC-Aβ1-42 peptides in murine microglia in response to antiretroviral compounds. In contrast, Brown et al. [39] reported that AZT and 3TC alone did not reduce FITC-Aβ1-42 phagocytosis in primary mouse microglia. In an experimental study involving adult male Wistar rats treated with 3TC (6 mg/kg), a substantial increase in the microglial activity was observed, as evidenced by a significant upregulation of reactivity for CD68. This heightened activation of microglia suggests an intensified phagocytic activity in response to pronounced neuroinflammation induced by 3TC [40]. Liuzzi et al. [41] conducted experiments on primary cultures of rat microglia treated with various doses of AZT for 20 h and simultaneously activated via an exposure to lipopolysaccharide. The assessment of the culture supernatants collected from the microglia did not reveal an increase in the MMP-2 mRNA and protein expression in response to LPS or combined AZT and indinavir treatment. While the LPS treatment induced the expression of MMP-9, it was dose-dependently inhibited by AZT and indinavir treatment in the LPS-stimulated microglia. Furthermore, there is supportive evidence suggesting that the pathogenesis of HIV-associated dementia is likely attributed to indirect effects of HIV infection on the brain, possibly mediated through the actions of macrophages and microglia [42]. Faria et al. [43] proposed the utilization of lipid nanocarriers for anti-HIV therapeutics to enhance penetration into HIV reservoir sites and surmount biological barriers such as BBB upon administration. Nanocarriers, when coated with specific surface stabilizers, offer potential utility in achieving elevated drug concentrations in the brain during CNS administration. The capacity of lipid nanocarriers to facilitate the brain delivery of anti-HIV drugs has been extensively documented, with liposomes, in particular, demonstrating the potential to enhance the brain accumulation of AZT [44,45].

## 4. Materials and Methods

### 4.1. Cell line and Conditions

The HMC3 adherent cell line, which possesses characteristics akin to primary microglial cells, was procured from the American Type Culture Collection (CRL-3304). These cells were cultivated in either 25 cm^2^ or 75 cm^2^ culture flasks under controlled conditions at 37 °C with 5% CO_2_ in Eagle’s Minimum Essential Medium (ATCC 30–2003). The culture medium was supplemented with 2 mM L-glutamine, 1 mM sodium pyruvate, 1500 mg/L sodium bicarbonate, 10% fetal bovine serum (FBS), and 1% penicillin-streptomycin. To maintain cell viability and optimal growth, the culture medium was replenished every 2–3 days. When the cells reached 80–90% confluence, they were subcultured using TrypLE^TM^ Express Enzymesolution (12604013) from Gibco, Thermo Fisher Scientific, Waltham, MA, USA.

### 4.2. Tested Compounds

The concentrations of lamivudine (Sigma-Aldrich Saint Louis, MO, USA, Y0000425) and zidovudine (Merck, cat. nr. Z1900000) selected for this study were chosen to align with the therapeutic concentrations achieved in blood serum during drug treatment—lamivudine at 10 μM and zidovudine at 6 μM, as previously established [46,47,48]. In accordance with the FDA’s National Drug Code, the following abbreviations will be used: 3TC for lamivudine and AZT for zidovudine. Stock solutions of 3TC and AZT were prepared by dissolving the substances in phosphate-buffered saline (PBS). To exemplify the effects of saturated fatty acids, palmitic acid (PA) was selected for the study. The following concentrations of PA were chosen to represent both physiological and elevated levels of free fatty acids (FFAs) in blood serum: palmitic acid at a physiological concentration of 200 μM and at a lipotoxic concentration of 500 μM [49]. For the conjugation of PA with fatty acid-free bovine serum albumin (BSA, Sigma-Aldrich Saint Louis, MO, USA, A7030), a three-step process was employed. Initially, 1 g of PA powder was dissolved in 7.8 mL of 99% ethanol to create a 500 mM solution at 37 °C. Subsequently, this solution was filtered using a 0.2 μm sterile filter. In the next step, 1.5 g of fatty acid-free BSA was diluted in 15 mL of serum-free media at 37 °C and also filtered using a 0.45 μm sterile filter. Finally, a 5 mM PA-BSA solution was obtained by combining these two solutions at a 100:1 ratio (BSA:PA) [50]. A solution of the positive control, cis-4,7,10,13,16,19-docosahexaenoic acid (DHA, Sigma-Aldrich, catalog number D2534), was prepared using the same protocol and used in concentrations equivalent to those of PA.

### 4.3. Cell Cytotoxicity Assay

Cell viability following treatment with DHA, PA, 3TC, or AZT was assessed using a colorimetric XTT assay (Roche Basel, Switzerland, 11465015001). To initiate the assay, 4 × 10^4^ cells were seeded into wells in a 96-well plate. After a 24 h incubation period, allowing for cellular attachment, the culture medium was replaced, and the specified concentrations of PA, DHA, 3TC, or AZT were introduced to the cells. The incubation was carried out for 24 h at 37 °C, under conditions of 5% CO_2_ and 95% humidity. A freshly prepared XTT mixture was employed for the assay, created by blending the XTT labeling reagent with the electron coupling reagent at a ratio of 50:1. Subsequently, 50 µL of the XTT mixture was added to the cells, and the cells were further incubated for 18 h at 37 °C, maintaining an environment of 5% CO_2_ and 95% humidity, adhering to the manufacturer’s protocol. Following this incubation period, the absorbance of the samples was determined at 450 nm, with reference measurements taken at wavelengths exceeding 650 nm. This analysis was conducted utilizing a Synergie multi-well scanning spectrophotometer (STAT FAX 2100, Awareness Technology, Inc., Palm City, FL, USA).

### 4.4. Intracellular ATP Test

Intracellular ATP levels were quantified by employing the ATP Bioluminescence Assay Kit HS II (Roche, 11699709001) following the manufacturer’s protocol. Concisely, HMC3 cells were seeded at a density of 4 × 10^4^ cells per well in 96-well plates and allowed to adhere for 24 h. Subsequently, they were exposed to the designated concentrations of PA, DHA, 3TC, or AZT for a 24 h incubation period. Upon completion of the treatment, the cells were lysed, and luciferase reagent was introduced into both the experimental samples and standards. The resulting green luminescence was quantified utilizing a luminometer (Spark^®^ multimode microplate reader, Tecan, Männedorf, Switzerland) with measurements taken at 562 nm after a brief 1 s delay and integrated over the span of 1 to 10 s. To derive the actual ATP concentrations, blank readings were subtracted from the raw data, and the values were calculated using a log–log plot based on the standard curve data.

### 4.5. Levels of Mitochondrial Superoxide 

The assessment of mitochondrial superoxide levels was conducted using the MitoSOX^TM^ Red assay (Thermo Fisher Scientific Waltham, MA, USA, M36008) in accordance with the manufacturer’s instructions. In brief, a 5 mM stock solution of MitoSOX^TM^ reagent was freshly prepared by dissolving the contents of the vial in 13 µL of anhydrous dimethyl sulfoxide (DMSO). A working solution of 500 nM was created by adding 5 µL of the stock solution to 50 mL of phosphate-buffered saline (PBS). HMC3 cells were seeded at a density of 4 × 10^4^ cells per well in 96-well plates and allowed to adhere for 24 h. Subsequently, they were subjected to specified concentrations of PA, DHA, 3TC, or AZT for a 24 h incubation period. Following the incubation, supernatants were collected, and the HMC3 cells were gently washed with room-temperature PBS. The cells were then fixed with 100% cold methanol for 5 min at 4 °C. After fixation, the cells were rinsed three times with PBS, and 100 µL of the MitoSOX^TM^ reagent working solution was added to each well. The cells were incubated in the dark at 37 °C for 10 min. Following incubation, the HMC3 cells were washed once with PBS, and the fluorescence was measured for the entire well using a Spark^®^ multimode microplate reader from Tecan, Männedorf, Switzerland (excitation at 510 nm and emission at 560 nm).

For fluorescence imaging, 4 × 10^4^ HMC3 cells were seeded in 48-well plates and treated as described above. Following methanol fixation, the cells were counterstained with diamidino-2-phenylindole (DAPI) for 15 min, and the mitochondrial superoxide production was visualized using a fluorescence microscope (CKX53, Olympus, Hamburg, Germany.

### 4.6. Protein Concentration Assessment

The Pierce™ BCA Protein Assay Kit (Thermo Fisher Scientific, Waltham, MA, USA 23227) was employed to quantify the total protein concentration in the samples. The working range for total protein measurement spanned from 5 to 250 ng/mL. Cell pellets from a 96-well plate, each containing 4 × 10^4^ cells, were spun down, and 10 µL from each cell pellet was pipetted into a well of a microplate. Subsequently, 200 µL of the working reagent was added to both the samples and standards. The plate was then placed on a plate thermostatic shaker (DTS-2, Elmi SIA, Riga, Latvia) and mixed for 30 s. Following the mixing step, the plate was covered and incubated at 37 °C for 30 min, after which it was allowed to cool to room temperature. The absorbance was measured at 562 nm using a Synergie multi-well scanning spectrophotometer (STAT FAX 2100, Awareness Technology, Inc., USA), and the total protein concentration was determined based on a standard curve.

### 4.7. FABP4 and FABP5 Concentrations

The intracellular concentrations of fatty-acid-binding protein 4 (FABP4) and 5 (FABP5) were quantified through the utilization of enzyme-linked immunosorbent assays. The Human FABP4 ELISA Kit (ab234565, Abcam, Cambridge, UK) and the Human FABP5 ELISA Kit (E1399Hu, Bioassay Technology Laboratory, Birmingham, UK) were employed for these measurements. HMC3 cells were plated in 96-well plates at a density of 4 × 10^4^ cells per well and allowed to adhere for 24 h. Subsequently, they were exposed to the respective concentrations of DHA, PA, 3TC, or AZT for a 24 h duration. Following the treatment, the cells were lysed using radioimmunoprecipitation assay buffer. The subsequent FABP4 and FABP5 ELISA assays were conducted following the guidelines provided by the manufacturers. The cellular pellet was standardized for cellular protein concentration using the BCA assay, and the protein concentration was measured. Adjustments were made to ensure that the protein concentration in the cell lysates was maintained at 100 µg/mL total protein concentration.

### 4.8. MAPK Family Activation Measurement

The intracellular activation of ERK1/2, p38, and JNK1/2 was assessed in HMC3 cell lysates using the InstantOne ELISA™ kit (IOAP96, Thermo Fisher Scientific, Waltham, MA, USA). The ELISA test was executed in accordance with the provided manufacturer’s protocol. HMC3 cells were seeded in 96-well plates at a density of 4 × 10^4^ cells per well and allowed to adhere for 24 h. Subsequently, they were exposed to the respective concentrations of DHA, PA, 3TC, or AZT for a 24 h period. Following treatment, the cells were lysed using RIPA buffer. The extent of activation/phosphorylation of ERK1/2, p38, and JNK1/2 proteins in HMC3 cell lysates was quantified, with reference to both negative and positive control samples.

### 4.9. Lipid Concentration and Staining Protocol

The HMC3 cells, incubated for 24 h with either NRTIs or fatty acids, underwent Oil Red O staining (00625-25G, Sigma Aldrich, Saint Louis, MO, USA) to assess the accumulation of lipid droplets. The Oil Red O working solution was meticulously prepared by adhering to the manufacturer’s instructions.

For lipid quantification, HMC3 cells were initially seeded at a density of 4 × 10^4^ cells per well in a 96-well plate. Subsequently, they were left undisturbed for 24 h to ensure proper adherence before being exposed to varying concentrations of DHA, PA, 3TC, or AZT for a 24 h duration. Following the treatment period, the cells were fixed with 4% paraformaldehyde for 1 h at room temperature. Subsequent to fixation, the cells were incubated with 0.5% Triton X100 in PBS for 30 min at room temperature, washed thrice with PBS, and incubated with the Oil Red O working solution for 2 h. After Oil Red O staining, the cells underwent three PBS washes and were air-dried at 32 °C. To extract and dissolve the Oil Red O that had adhered to the HMC3 cells, 100 µL of isopropanol was added to each well. Then, 100 µL of this extract was transferred to fresh wells, and the absorbance at 510 nm was measured using a Synergie multi-well scanning spectrophotometer (STAT FAX 2100, Awareness Technology, Inc., Palm City, FL, USA).

For microscopic evaluation, HMC3 cells were seeded in 6-well plates at a density of 5 × 10^5^ cells per well and allowed to adhere for 24 h. Subsequently, they were treated with various concentrations of DHA, PA, 3TC, or AZT for 24 h. Following the treatment period, the cells were fixed with 4% paraformaldehyde for 1 h at room temperature. After fixation, the cells were incubated with 0.5% Triton X100 in PBS for 30 min at room temperature, followed by three PBS washes. The cells were then incubated with the Oil Red O working solution for 2 h. Following Oil Red O staining, the cells underwent three PBS washes and were counter-stained with DAPI for 15 min. The lipid droplets were visualized using an inverted microscope (CKX53 Olympus, Hamburg, Germany).

### 4.10. Statistics

Statistical analysis was conducted by employing parametric tests, specifically ANOVA, along with the application of appropriate post hoc tests. These statistical methods were chosen due to the normal distribution of the data and the equality of variance within the dataset. The results are expressed in the format of mean ± standard deviation (SD). The statistical analysis was executed using Statistica 13.1 software by Dell Software Inc., Port St. Lucie, FL, USA. For each experimental condition, a minimum of three independent experiments were performed to ensure the reliability of the results. The descriptive statistics are presented as mean ± SD. Significance levels are indicated as follows: * *p* < 0.05; ** *p* < 0.01; *** *p* < 0.001.

## 5. Conclusions

In the context of the CNS, it is important to note that HIV primarily resides within the cerebral microglia or macrophages, which are the innate immune cells in the brain parenchyma, as opposed to astrocytes, as observed in the brains of HIV-1-infected aviremic individuals undergoing suppressive antiretroviral therapy [42]. The various cellular reservoirs for HIV-1 within the CNS may play crucial roles in the molecular mechanisms associated with HIV-1 neuropathogenesis. In summary, this study sheds light on the potential impact of nucleoside reverse transcriptase inhibitors (NRTIs) on the development of lipotoxicity in neuronal cells. However, it is imperative to recognize that the observed effects may vary according to the duration of exposure to these compounds. Lipid toxicity appears to have been induced in microglial cells by the tested drugs, although the extent of this effect was insufficient to trigger cell death. Nevertheless, it is essential to acknowledge the various limitations of this research. One key limitation is that the study was conducted on an in vitro cell line, which means that the results obtained here may not perfectly mirror the responses observed in patient-derived materials. The microenvironmental conditions in vivo and the influence of numerous other factors may yield different outcomes. Additionally, the relatively short-term exposure of cultured cells to these drugs contrasts with the prolonged drug regimens taken by patients, thereby potentially leading to varying consequences. To gain a more comprehensive understanding of the cellular changes associated with lipid metabolism and to confirm the link between these changes and the mitochondrial dysfunction and reactive oxygen species (ROS) production induced by the drugs, as postulated by several researchers, future investigations should include assessments of ROS concentrations, lipid peroxidation, and the presence of other oxidative stress markers. The subject of lipid disorders within the CNS under the influence of NRTIs remains an area of inquiry with many unanswered questions. Therefore, it is imperative to continue researching in order to unravel the intricate mechanisms behind this phenomenon. Such knowledge is of paramount importance as it holds the potential to mitigate the risks of complications related to lipotoxicity in patients undergoing NRTI therapy. Further research endeavors are warranted to provide a more comprehensive understanding of the intricate interplay between NRTIs and lipid metabolism within the CNS.

## Data Availability

Data are contained within the article.
